# Endocannabinoids and Schizophrenia

**DOI:** 10.3390/ph3103101

**Published:** 2010-10-08

**Authors:** Joëlle Desfossés, Emmanuel Stip, Lahcen Ait Bentaleb, Stéphane Potvin

**Affiliations:** Centre de Recherche Fernand-Seguin, Department of Psychiatry, University of Montreal, 7331 Hochelaga, Montreal, Quebec, H1N 3V2, Canada; E-Mail: joelle.desfosses@umontreal.ca (J.D.); emmanuel.stip@umontreal.ca (E.S.); lahcen.ait.bentaleb@umontreal.ca (L.A.B.)

**Keywords:** endocannabinoids, schizophrenia, cannabis, drug abuse, metabolism

## Abstract

The endocannabinoids anandamide and 2-arachydonoylglycerol (2-AG) are lipids naturally derived from membrane precursors which bind cannabinoid receptors (CB_1_, CB_2_). This endocannabinoid system is disturbed in schizophrenia. Indeed, there seems to be an association between schizophrenia and polymorphisms of the CB_1_ receptor gene. Moreover, CB_1_ receptors are found in higher density in the prefrontal cortex, hippocampus and basal ganglia of patients with schizophrenia. Similarly, anandamide levels are increased in the cerebrospinal fluid (CSF) and in the serum of schizophrenia patients, including during the prodromal state, suggesting that they may play a protective role in psychosis homeostasis. Future studies are needed to further explore the role of the endocannabinoid system in the pathophysiology of schizophrenia.

## 1. Introduction

Schizophrenia is a complex psychiatric disorder with a lifetime prevalence of 0.4% in the general population [1,2,3] Thus far, several etiological models have been proposed to explain the biological bases of the disorder, including neurodevelopmental, neurodegenerative or cortical-subcortical disconnection models. Admittedly, schizophrenia is associated with several comorbidities, encompassing the fields of addiction, endocrinology, immunology and neurology. It is of great interest to explain this aggregation of signs and symptoms from a neurobiological perspective. A disturbance of the cannabinoid system could contribute to the general understanding of the biological bases of schizophrenia and it may also be involved in its associated comorbidities. Here, we will review the literature about the endocannabinoid system, its biological roles and its interactions with neurotransmission systems, and we will subsequently evaluate its potential implication in the pathophysiology of schizophrenia and its associated comorbidities.

## 2. Cannabis and Schizophrenia

Patients with schizophrenia are more prone to substance abuse than the general population [4]. Among them, 25% have a lifetime prevalence of cannabis abuse/dependence, the most widely used illicit psychoactive substance [5]. Cannabis use disorder has a negative impact on response to antipsychotics, drug compliance and psychotic relapse. In regular users, cannabis induces euphoria, perceptual illusions, tachycardia, analgesia, memory and concentration alterations, and other cognitive deficits. Cannabinoid intoxication can provoke toxic psychoses or symptoms similar to the positive symptoms of schizophrenia and even the pathognomonic schneiderian psychotic symptoms (e.g., thought intrusion/diffusion). In a randomized, double-blind, placebo-controlled study, intra-venous Δ^9^-THC (the main psychoactive agent of cannabis) administered to human healthy controls produced positive (delusions and hallucinations) and negative symptoms (blunted affect and social withdrawal), as well as cognitive effects, suggesting that it can be used as a valid model of psychosis [6]. Similarly, inhaled Δ^9^-THC has been shown to impair verbal memory, selective and sustained attention in both healthy subjects and schizophrenia patients [7]. Chronically, cannabis seems to provoke an amotivational syndrome similar to the negative symptoms of schizophrenia [8]. Therefore, cannabis can be used as a psychosis model, because its effects are more representative of the full spectrum of schizophrenia symptoms than the amphetamine sensitization model. Importantly, the psychotomimetic effects Δ-9-THC may not be solely mediated by dopamine-D_2_ receptor mechanisms in humans. Indeed, healthy subjects received haloperidol before receiving intra-venous Δ^9^-THC, but haloperidol pre-treatment failed to antagonize the psychotomimetic effects of Δ^9^-THC [9]. Further studies are therefore needed to better understand the link between cannabinoids and psychosis. 

Large longitudinal and cross-sectional studies have evidenced that cannabis smoking is a risk factor of psychosis [10]. In addition, cannabis smoking can exacerbate psychosis in schizophrenia patients [11]. Prospective studies revealed that cannabis consumption seems neither a sufficient nor a necessary cause for psychosis [12]. Its influence appears more complex and it may interact with many other factors to produce psychosis. From a vulnerability-stress perspective, it has been shown that the risk of developing schizophrenia-spectrum disorders is more elevated in cannabis smokers carrying the Val-Val genotype of the catechol-*O*-methyltransferase (COMT) Val158Met gene polymorphism [13]. COMT is an enzyme degrading catecholamines (dopamine and norepinephrine) in the frontal lobe, and the val-val genotype is associated with enhanced vulnerability for psychosis. 

Apart from these genetic influences, it seems that there is a dose-dependent relationship between the amount of cannabis used during adolescence and the subsequent risk of developing schizophrenia [14]. Such findings have been viewed as evidence that cannabis would be an independent risk factor for the emergence of psychosis in individuals without a psychosis background [15]. In this vein, it has been shown that a young age of commencement of substance abuse increases the risks of psychotic outcomes [16]. Consistently with the neurodevelopmental model of schizophrenia, it is also possible that exogenous cannabis smoking influences the neurodevelopmental processes thought to lead to schizophrenia [17].

These complex clinical, epidemiological and longitudinal relationships between schizophrenia and cannabis suggest that dysfunctions of the endogenous cannabinoid (ECB) system could be intrinsically involved in the pathophysiology of schizophrenia and some of its associated comorbidities.

## 3. Endocannabinoid System

The term cannabinoid encompasses all substances structurally related to cannabis. The ECB system includes ECB ligands, which are substances synthesized from lipid precursors in the neuronal membrane, which are part of the ethanolamine family. There are two main ECBs, *N*-arachidonoyl ethanolamide (anandamide) and 2-arachidonoyglycerol (2-AG) and two main cannabinoid receptors, CB_1_ and CB_2_ [18]. 

### 3.1. Receptors

The cannabinoid system includes two major receptors: CB_1_ and CB_2_. Cloned in 1990 [19], CB_1_ receptors are the most abundant G protein-coupled receptors in the central nervous system (CNS). In fact, they have a density 10 to 50 times greater than that of dopaminergic and opioidergic receptors. ECBs have a high affinity for CB_1_ receptors, which are located in the pre-synaptic neurons. They are found mainly in the CNS in brain areas such as the globus pallidus, the hippocampus, the cerebral cortex, the hypothalamus, the cerebellum, the striatum, and the mesencephalic **periaqueductal** gray matter [18]. Accordingly, ECBs are involved in brain functions such as pain, emotions, cognition and motivation [20]. CB_1_ receptors are also found in the periphery in reproductive, metabolic, cardiovascular and gastro-intestinal systems.

CB_2_ receptors were first identified on spleen macrophages [21]. They are mostly located in the periphery, mainly in immune cells, and they can modulate immune cell migration and cytokine release in periphery and in the brain [22,23,24,25]. They are notably highly expressed on B-cells and moderately found on monocytes and polymorphonuclear neutrophils, even though they are also found in the CNS on glial cells and brainstem and cerebellar neurons [26,27]. The evolution of knowledge about cannabinoid receptors revealed that both of them control central and peripheral functions such as cellular functions, neuronal development, neurotransmission, inflammation, cardiovascular, respiratory, reproductive and hormonal functions, energy metabolism and antinociception [23,27,28,29,30,31,32,33]. However, the exact role of neuronal CB_2_ receptors remains incompletely established. 

### 3.2. Ligands

Anandamide was the first discovered ECB [34]. It is considered a full agonist of CB_1_ receptors [35] and a partial agonist of CB_2_ receptors [36]. Anandamide has various roles in the CNS, including drug reward, memory and pain relief [18]. 2-AG is the second endogenous ligand of the cannabinoid system to be discovered [37]. It seems to have a better intrinsic efficacy on CB receptors [38] and more important effects on neurotransmission than anandamide. Indeed, 2-AG is a CB_1_-selective agonist compared to anandamide, which is an agonist without CB receptor selectivity [24]. As mentioned in the article, anandamide may affect neurophysiologic processes by interacting with other targets. In this perspective, 2-AG is increasingly considered the main ECB the recent literature. Elevation of anandamide by pharmacologic inhibition of its degradation enzyme, fatty acid amid hydrolase (FAAH), does not influence CB_1_ receptor activity [39]. There is also an interaction between anandamide and 2-AG. In fact, anandamide inhibits 2-AG rather than competing with CB_1_ receptors [40]. This finding raises the hypothesis that anandamide may affect neurophysiologic processes by interacting with other targets.

More recently, new ECBs have been identified, namely: *O*-arachidonoylethanolamine (virodhamine), arachidonoyldopamine [41] and noladin ether [42]. Virodhamine is considered a partial agonist of CB_1_ receptors and has possibly agonist-antagonist effects on these receptors [41]. As for palmithylethanolamide (PEA) and oleylethamolamide (OEA), these fatty acid ethanolamines involved in metabolism are considered endogenous ligands without bearing the cannabinoid denomination, because they have no effect on cannabinoid receptors (according to present knowledge) [43]. 

### 3.3. Other Receptors

Among other ECB targets, there are surface non-cannabinoid receptors, ion channels receptors and nuclear receptors. Recently, GPR55, a new orphan G protein-coupled receptor has been identified as a cannabinoid receptor [44,45], which favours neuronal excitability. Moreover, it has also been discovered that anandamide is an endogenous activator of the transient receptor potential, vanilloid sub-type, TRPV1 receptor [46,47]. TRPV1 is activated by inflammatory factors (such as nerve growth factors), and it is found on non-neuronal cells, as well as on dopamine neurons in the substantia nigra, on pyramidal neurons in the hippocampus, the locus coeruleus and several cortical layers. There is a specific interaction between anandamide and an intracellular site of TRPV1 [48]. The effect of anandamide on this channel can be diminished or blocked by specific antagonists of TRPV1, whereas it is not affected by antagonists of CB receptors. Noteworthy, 2-AG does not activate TRPV1 receptors. Interestingly, the action of ECBs on dopamine transmission may be mediated via TRPV1 receptors. Anandamide and analogs (AM404, NADA) are full TRPV1 agonists, while Δ^9^-THC does not bind vanilloid receptors. This latter finding could explain why anandamide decreases striatal dopamine release, whereas Δ^9^-THC increases it [46], given that *in vitro* and *in vivo* evidence tend to demonstrate that TRPV1 receptor activators inhibit dopamine release [49]. 

Although they are lipophilic, cannabinoids interact with nuclear receptors, such as peroxisome proliferator-activated receptors (PPARs), a family divided in three sub-types α, δ (or β), γ, all expressed in the nervous system [50,51]. Anandamide activates PPAR α and γ subtypes. In addition, OEA, PEA and anandamide have a high affinity for the binding site of PPAR, which confer them anorexigenic, anti-inflammatory, neuroprotective, anti-seizure, arousing, cognitive enhancing and anti-addictive properties [52].

### 3.4. General Functioning of the System

ECBs have a major function of synaptic communication regulation, through activation of CB_1_ receptors. ECBs are synthesized on request by neurotransmitters and are considered, like autacoids, local mediators similar to prostaglandins. Most neurotransmitters are released from pre-synaptic neurons and cause cellular depolarization or stimulate receptors in a calcium-dependent manner. ECBs are synthesized in post-synaptic neurons and released in synaptic cleft and then bind CB_1_ receptors and inhibit neurotransmitter release via retrograde signalling [53,54]. ECBs are then quickly degraded by hydrolysis in the post-synaptic and pre-synaptic neurons [55]. The integral membrane protein, FAAH, is the principle enzyme responsible for anandamide degradation in the CNS [56]. FAAH terminates this lipid signalling by hydrolyzing it to arachidonic acid and ethanolamine. For 2-AG, the main degradation enzyme in the CNS is monoacylglycerol lipase [57]. 

In the brain, the ECBs interact with neurotransmission systems involved in the pathophysiology of schizophrenia, such as dopamine, gamma-amino butyric acid (GABA), acetylcholine and glutamate [58]. ECBs mainly modulate neurotransmission in pre-synaptic neurons, where CB_1_ receptors are located. CB_1_ and D_2_ receptors are highly co-expressed in the striatum, and produce opposite effects on the regulation of locomotion in mice [59]. In addition, dopamine causes transient calcium-dependent release of ECBs in ventral tegmental area and anandamide can inhibit dopamine release in the striatum, as a retrograde messenger. In contrast, exogenous cannabinoids such as Δ^9^-THC and synthetic CB_1_ agonists increase dopamine synthesis in the nucleus accumbens and prefrontal cortex [60]. This mechanism may underlie the increased risk for psychosis in cannabis smokers as well as the exacerbation of psychotic symptoms by cannabis smoking in schizophrenia patients [11].

CB_1_ receptors are located on glutamatergic projections to the neo-cortex, the hippocampus, the hypothalamus and the cerebellum, as well as on ascending cholinergic, serotoninergic, noradrenergic subcortical pathways [61,62,63,64]. They are also located on GABAergic interneurons containing cholecystokinin (CCK) [65]. CB_1_ receptors inhibit GABA, glutamate, acetylcholine and norepinephrine release [20]. Additionally, CB_1_ receptors play a major role on GABAergic interneurons in the hippocampus, which are engaged in synchronisation of neuronal activity [66,67]. In general, ECBs are produced after intense neuronal activity [68] and are involved in synaptic plasticity, long-term potentiation and long-term depression, particularly in the hippocampus, where they may alter cognitive functioning and sensory gating.

### 3.5. Endocannabinoids and neurogenesis

ECBs influence neurodevelopmental processes, such as neuronal specification, migration and maturation, axonal elongation and synaptogenesis [69]. CB_1_ receptors are more abundant in the white matter of the embryonic brain while levels increase in the grey matter from the prenatal period up to adult age. This expression is correlated with progression of neuronal differentiation [70], leading to distribution of CB_1_ receptors in cortical layers I to VI under the influence of excitatory neurons vGlut-1 [71] and GABAergic interneurons containing CCK [72,73]. Theoretically, the cell fate could be disturbed by exogenous cannabinoids during adolescence or, even sooner, by a prenatal exposure, possibly leading ultimately to psychotic disturbances. 

## 4. Endocannabinoids and Schizophrenia

Schizophrenia is a complex disorder that many theories fail to fully explain. The current literature focuses on dopaminergic dysfunctions, although other neurotransmitters are thought to be involved, including serotonin, acetylcholine and glutamate [74]. During the last decade or so, animal and human studies have both provided converging evidence suggesting strong links between schizophrenia and cannabinoids. The ECB system plays an active role in brain regions disturbed in schizophrenia and interacts with the main neurotransmitters thought be involved in the pathophysiology of schizophrenia. Accordingly, mounting evidence suggests that the ECB system is dysfunctional in schizophrenia.

### 4.1. CB1 receptors - genetics, post-mortem and in vivo studies.

CB1 receptor gene (*CNR1*) encodes the CB_1_ receptor and is located on chromosome 6q14–15, which has been considered as a susceptibility locus for schizophrenia [75]. A repetition of nine AAT triplets of the polymorphism in the 3’ flanking region of *CNR1* gene was associated with a susceptibility to develop the hebephrenic subtype of schizophrenia, which is characterized by prominent disorganization and negative symptoms [76]. Interestingly, the schizophrenia symptoms associated with the AAT repeat marker of the *CNR1* gene are similar to those observed in chronic cannabis-induced psychosis [77]. Additionally, a biallelic single-base polymorphism within the first exon of the *CNR1* gene, consisting of a silent mutation of 1359 G-to-A in the 453 codon (threonine) has been discovered [78]. It has been explored in patients with schizophrenia. No significant difference was observed in the allele frequency or genotype distribution between patients and controls [79]. However, there was significantly less frequent homozygote GG genotypes in non-abusing patients compared to substance-abusing schizophrenia patients, and there were no differences between the latter group and healthy controls. Thus, the current state of the literature does not suggest that the 1359G/A polymorphism would be a vulnerability factor for schizophrenia, as it seems, rather, to influence antipsychotic response and side effects. For instance, the G allele of the 1359G/A polymorphism of the *CNR1* gene was found in excess in patients with schizophrenia refractory to atypical antipsychotics and the highest non-responsiveness was found in patients with the homozygous GG genotype [80]. Similarly, the rs806378 single nucleotide polymorphism (SNP) of the *CNR1* gene was recently associated with antipsychotic-induced weight gain in a small population of chronic schizophrenia patients [81]. Overall, the current available evidence does not indicate direct associations between *CNR1* variations and susceptibility to schizophrenia [82]. Moreover, an association was recently observed between schizophrenia and two SNPs in and near the CB_2_ receptor gene (*CNR2*): rs12744386 and rs2501432. In patients with schizophrenia, there was a significant increase in the frequency of the R63 allele of rs2501432 (R63Q) polymorphism as well as the C allele of rs12744386 polymorphism. This study suggested that the susceptibility for schizophrenia is increased by a genetically predetermined lower functioning of CB_2_ receptors [83]. 

CB_1_ receptors are dense in several brain regions disturbed in schizophrenia, such as the hippocampus and the basal ganglia. Post-mortem human brain studies have shown an increase in CB_1_ receptor density particularly in the dorsolateral prefrontal cortex of patients who suffered from schizophrenia in their life, without regards to cannabis smoking [84]. Among subjects who had used cannabis shortly before death, there was an increase in CB_1_ receptors density in the caudate-putamen, independently of schizophrenia. Knowing the significant CB_1_ receptor density in the prefrontal cortex, another post-mortem study assessed CB_1_ receptor *mRNA* levels and found that they were 14% lower in the dorsolateral prefrontal cortex of schizophrenia patients, relative to healthy controls. Similarly, a post-mortem study showed an increase in CB_1_ receptor density in the anterior cingulate cortex of schizophrenia patients, compared to healthy subjects [85]—a result that was not confirmed using immunohistochemistry [86]. Knowing that the functioning of the posterior cingulate cortex (PCC) may also be altered in schizophrenia [87], it is also intriguing to notice that the densities of CB_1_ and CB_2_ receptors are elevated in the PCC of schizophrenia patients, relative to matched controls [88]. Finally, another post-mortem study showed that the frontal CB_1_ receptor density in frontal cortex was decreased among schizophrenia patients treated with antipsychotics, compared to untreated patients [89]. Assuredly, there are many confounding factors influencing the results of post-mortem studies, such as antipsychotic treatment, cannabis smoking and the heterogeneity of biochemical techniques. However, the preliminary results from these studies suggest that CB_1_ receptor functioning may be altered in schizophrenia in brain regions involved in cognition [90], which is prominently impaired in schizophrenia [91].

A recent study has brought new insights about the link between schizophrenia and CB_1_ receptors. With a novel positron emission tomography (PET) radioligand, [11C] OMAR (JHU75528), it was possible for the first time to examine *in vivo* CB_1_ receptor binding. In this PET study, there was an elevation of CB_1_ binding in patients with schizophrenia across many brain regions, relative to healthy controls [90]. However, the only significant binding elevation was reported in the pons.

In animal models of schizophrenia such as social or maternal deprivation, significant changes in ECB signalling have been reported, including a significant decrease in CB_1_ receptor expression in the caudate putamen and the amygdala and a significant increase in FAAH expression in the caudate putamen and the nucleus accumbens [92]. There was no significant change in D_2_ receptor expression in any region studied in this experimental investigation, indicating that the ECB system is altered in animal models of psychosis. Noteworthy, another preclinical study examined ECB levels and CB_1_ receptor binding in a pharmacological model of schizophrenia-like cognitive deficits (e.g., withdrawal from subchronic administration of phencyclidine –PCP– in rats). PCP-treated rats showed increased ECB levels in the nucleus accumbens and ventral tegmental area, whereas CB_1_ receptor expression was unaltered. This study suggested also an interesting element: FAAH inhibition or CB_1_ receptor blockade may improve negative symptoms in PCP-treated rats but produce deleterious effects in untreated animals, possibly by disturbing ECB tone [93]. Moreover, another study using the same pharmacological model showed that in the prefrontal cortex of PCP-treated rats, there was a significant reduction in CB_1_ receptor binding and an increase in 2-AG levels, suggesting that prefrontal dysfunctions of ECB system could contribute to the glutamatergic-related cognitive deficits in schizophrenia [94]. 

Moreover, in genetic studies on knock-out mice for CB_1_ and CB_2_ receptors genes, psychotic-like behavioural changes were observed. CB_1_ receptor knock-out mice exhibited behavioural alterations reminiscent of schizophrenia, of cannabinoid intoxication and of dopamine-related behaviours. In animal studies, ECBs have also been shown to play a protective role against psychotic-like behavioural disturbances, especially stereotypies. Indeed, it was shown that the motor responses produced by amphetamines and by quinpirole (a D_2_ receptor agonist) are enhanced after desensitization of the cannabinoid CB_1_ receptor [95,96], suggesting that ECB downregulation can lead to sensitized psychotic-like states.

### 4.2. Endogenous ligands

There is accumulating evidence of anandamidergic dysfunction in schizophrenia. Actually, CSF anandamide levels are significantly elevated in schizophrenia patients, compared to healthy controls and individuals with other psychiatric disorders such as depression, bipolar disorder, as well as Alzheimer’s disease and vascular dementia [97,98]. This elevation in anandamide levels is not only observed in the CSF, but also in the plasma. Among patients in the acute phase of schizophrenia, plasmatic anandamide levels were found to be significantly elevated, relative to healthy controls [99]. In this study, the *mRNA* plasmatic levels of the anandamide degradation enzyme, FAAH, were also elevated. Based on these findings, it has been hypothesized that an underlying compensatory mechanism elevates FAAH levels to normalize circulating anandamide levels. Similarly, plasmatic anandamide was found to be elevated in dual diagnosis patients with schizophrenia and substance abuse [100]. However, 2-AG levels were not elevated in these dual diagnosis patients, compared to controls, although this result possibly reflected a type-II error due to the size of the sample involved. 

### 4.3. Symptomatology

Given increasing evidence implicating the ECB system in the pathophysiology of schizophrenia, it becomes necessary to explore in more details the correlation between ECBs and the symptoms of schizophrenia. Psychotic symptoms arise when there is abnormal dopaminergic discharge in the mesolimbic system [74]. Functional interactions between cannabinoids and dopaminergic transmission have been suggested for many years [101]. For instance, dopamine-D_2_ receptor overactivity is associated with an increase of anandamide release in rodents [101]. In humans, a negative correlation has been found between CSF anandamide levels and the positive symptoms of schizophrenia [102]. As such, these results suggest that anandamide may normalize, in a compensatory fashion, the neurochemical disturbances associated with the acute phase of psychosis. In that perspective, the acute phase of psychosis would represent a failure of anandamidergic compensatory mechanisms. Noteworthy, recent data shows that this protective mechanism seems to be active before the apparition of acute psychotic symptoms. In fact, CSF anandamide has been shown to be elevated (not significantly) during the prodromal period of schizophrenia [102]. More precisely, that study showed that lower anandamide levels were associated with earlier transition time to acute psychosis [102]. In rodents, an acute CB_1_ receptor blockade with a selective antagonist potentiates stereotypy induced by D_1_ and D_2_ receptor agonists, suggesting that ECBs play inhibitory functions in the presence of dopaminergic overactivity [103]. Therefore, in the acute phase of the disorder, ECBs may counteract hyperdopaminergic disturbances. This observation contrasts with the fact that exogenous cannabinoids precipitate psychosis. This discrepancy could be explained by the pleiotropic effects of the ECBs, which depend on tissue type and on the stage of the disorder (acute or chronic), changing the functional outcomes of ECBs [104] Also noteworthy, ECBs interact with receptors different than Δ^9^-THC targets. At high dosage, ECBs interact with PPARs, leading to variable effects. In addition, Δ^9^-THC does not bind vanilloid receptors, contrarily to ECBs. As mentioned earlier, this latter finding could explain why anandamide decreases striatal dopamine release, whereas Δ^9^-THC increases it [46]. 

Additionally, the first PET study measuring CB_1_ receptor binding *in vivo* in schizophrenia highlighted an association between elevated CB_1_ binding in specific brain regions and the symptoms of schizophrenia [90]. More precisely, the highest elevated CB_1_ binding was correlated with the highest psychosis to withdrawal scores ratio in the frontal lobe and middle and posterior cingulate cortices. The preliminary results of this study suggest that there may be complex interactions between the positive and negative symptoms of schizophrenia and CB_1_ receptor functioning [90].

### 4.4. Endocannabinoids and the neurodevelopmental model of schizophrenia

From a physiologic perspective, ECBs have an essential role in neurogenesis, as previously mentioned. This knowledge raises the question of the potential role of ECBs in the neurodevelopmental model of schizophrenia. Although preliminary, emerging data are consistent with this view. For instance, neuregulin 1 and Erb4 receptor genes are involved in neurogenesis and are influencing the vulnerability to develop schizophrenia [105]. Interestingly, it has been shown that mice with hypomorphic neuregulin are more sensitive to the psychotic-like effects of Δ^9^-THC and that they display enhanced Δ^9^-THC-induced c-Fos expression [106,107]. Although this view is purely theoretical at the moment, it is also possible that ECBs play a role in the neurodegenerative model of schizophrenia. ECBs regulate neurotransmission and serve as a balance between excitatory and inhibitory neurotransmission, and a disturbance in the ECB system could possibly produce glutamatergic-mediated neurotoxic effects [58].

### 4.5. Antipsychotic perspectives

Cannabis-induced psychosis and schizophrenia have a good response to antipsychotic treatment. Considering the cannabinoid hypothesis of psychosis, we can suppose that the ECB system is not only involved in psychosis but also in the effects of antipsychotics. In human studies, clinical remission is accompanied by a significant reduction of anandamide levels following olanzapine treatment and also by reductions of CB_2_ *mRNA* and FAAH *mRNA* levels [99]. However, it must be considered that this trial included a small sample of patients. In a cross-sectional study, it was shown that schizophrenia patients treated with typical antipsychotics (D_2_ antagonists) had similar CSF anandamide levels, relative to healthy controls [98]. This result was interpreted as being consistent with preclinical data showing that D_2_ antagonism decreases ECB excess, while D_2_ agonism induces anandamide synthesis in limbic and motor areas. In contrast, schizophrenia patients treated with atypical antipsychotics (D_2_ and 5-HT_2_ antagonists) had elevated anandamide levels, as did drug-free schizophrenia patients. Thus, there was no normalisation of anandamide levels with atypical antipsychotics. 

Clozapine is an atypical antipsychotic potentially efficacious for the treatment of substance abuse, especially cannabis smoking, in schizophrenia patients [108]. In rats, clozapine influenced the binding of a selective CB_1_ agonist (CP,55940) in the nucleus accumbens after one and three months of treatment, a result that was not observed with other antipsychotics [109]. This effect was selective to the nucleus accumbens, a region thought to be dysfunctional in both substance abuse and psychosis, while no effect was observed in the hippocampus, the striatum and the frontal cortex. This result was not replicated in humans but comforts the idea that clozapine may be advantageous in dual diagnosis patients. The mechanisms underlying the effects of clozapine on CB_1_ receptors remain unknown. Given the interactions between CB_1_ and muscarinic receptors [110], it has been proposed clozapine could modify CB_1_ receptor binding via its effects on muscarinic receptors. Apart from these effects of clozapine, some typical antipsychotics (e.g., haloperidol and sulpiride) have been shown to enhance CB_1_ receptor *mRNA* levels in the striatum after one month of treatment [111].

Quetiapine is an atypical antipsychotic that shares several pharmacologic properties with clozapine, such as fast dissociation from D_2_ receptors, a similar affinity ratio for 5-HT_2A_ and D_2_ receptors, and a partial agonism at 5HT_1A_ receptors [112]. Thus, quetiapine and clozapine could have similar effects on CB_1_ receptors, since quetiapine has also demonstrated therapeutic benefits in dual diagnosis patients. However, in an open-label trial from our group, quetiapine did not affect plasma levels of anandamide and 2-AG in dual diagnosis patients following 12-week treatment [100]. However, substance abuse was a major confounding factor in that study.

Other pre-clinical studies with atypical antipsychotics found that risperidone increases CB_1_ binding in the rat caudate nucleus, hippocampus, and amygdala [113]. Moreover, olanzapine significantly decreases CB_1_ binding in the dorsal vagal complex of the brainstem in *ex vivo* experiments in rats while aripiprazole and haloperidol have little affinity for CB_1_ receptors [114]. However, both *in vitro* and *in vivo*, there is evidence that antipsychotics such as clozapine, olanzapine, haloperidol have no affinity for CB_1_ receptor [115]. Overall, even though antipsychotics may decrease CB_1_ radiotracer binding in the brainstem and amygdala by an unknown exact mechanism, the current evidence tends to suggest that antipsychotic drugs do not bind CB_1_ receptors *in vitro* and do not change the CB_1_ radiotracer binding in the cortex and striatum, where CB_1_ receptors have the highest density. 

Rimonabant (SR141716), a selective CB_1_ receptor antagonist, produces in rats changes in Fos protein expression and neurotensin in mesocorticolimbic areas that are a similar to those produced by atypical antipsychotics. Animal experiments also showed that rimonabant was able to reduce hyperactivity induced by psychostimulants, which can produce psychotic symptoms in humans [116]. Otherwise, a more recent pre-clinical study examined the effects of rimonabant (SR141716A) on prepulse inhibition (PPI) (e.g., the inhibition of a response to a strong startling stimulus by a weaker prestimulus), which is impaired in schizophrenia [117]. In rats, rimobanant administration counteracted PPI produced by the glutamatergic *N*-methyl-D-aspartate receptor antagonists, PCP and dizocilpine, and by the D_2_ agonist apomorphine, whereas SR141716 did not alter PPI when administered alone. Given that clozapine produced similar results on the PPI-disruptive effects of phencyclidine, dizocilpine and apomorphine, the authors interpreted their results as evidence of an atypical antipsychotic profile of rimonabant [118]. 

In humans, there is growing interest in the potential applications of the ECB system for the development of novel antipsychotic drugs. In that perspective, a functional magnetic resonance imaging study showed that 7-day administration of rimonabant (CB_1_ antagonist) to healthy participants reduced their subjective experience of reward (anhedonia) and their neural responses in key brain reward regions (e.g., ventral striatum) [119]. However, in a randomized placebo-controlled study involving adults with schizophrenia or schizoaffective disorder, rimonabant did not improve psychotic symptoms conclusively [120]. The second most abundant exogenous cannabinoid, cannabidiol (CBD), has anticonvulsant, antianxiety, antiinflammatory, neuroprotective and antiemetic properties as suggested by preclinical evidence [121,122]. However, it has no psychoactive properties. It is a weak partial antagonist at the CB_1_ receptor [123] but possibly acts also on new cannabinoid receptors [124,125], such as receptor GPR55 [126]. The effects of CBD could also be due to its inhibition of anandamide reuptake and enzymatic hydrolysis [121]. Favourably, Leweke and colleagues compared the antipsychotic activity of CBD with amisulpride in patients with acute schizophrenia, in a 4-week, double-blind controlled trial, presented in 2005 [118]. It revealed significant reductions of acute psychotic symptoms after 2 and 4 weeks of treatment with CBD (800 mg/day) that did not differ from amisulpride (800 mg/day) except for a lower incidence of side effects. 

In the end, ECBs’ actions depend on the functional balance between GABAergic and glutamatergic inputs that are both inhibited by ECBs under physiological conditions [104]. Interestingly, ECBs seem to produce effects that are dose-dependent and state-dependent [127]. Because of similarity of ECBs’ effects with the effects of partial agonists, it remains a challenge to predict the potential therapeutic utility of medications acting on this system.

## 5. Schizophrenia and its Associated Comorbidities

The ECB system represents a physiologic system allowing the organism to reach homeostasis, and as such, it could be involved in biological functions such as appetite regulation, food intake, metabolic regulation, weigh gain, learning/memory, pain avoidance and reward seeking. Knowing that schizophrenia is associated with metabolic disorders and increased vulnerability to alcohol and drug addiction, the ECB system seems to be implicated in these comorbidities. Consistent with this view, it is of interest that many of the CNR1 gene polymorphisms associated with schizophrenia have also been linked to metabolic disorders and substance abuse/dependence [128].

### 5.1. Endocannabinoids and metabolic control

Weight gain is influenced by various biological factors. Central mechanisms, such as food reward, have been shown to influence weight gain. The endocrinal system is also a critical player, as it is involved in both central and peripheral control of metabolism. Weight gain is also significantly influenced by peripheral mechanisms, such as metabolism and energy homeostasis. Interestingly, mounting pre-clinical evidence shows that the ECB system influences weight gain by acting at these various levels and plays a critical role in metabolic control and energy homeostasis [129]. 

At the CNS level, food reward has been shown to depend on CB_1_ receptors [130], given that CB_1_ antagonists block or diminish food reward. ECBs, more precisely anandamide, also influence food intake and weight gain by central actions on hypothalamic mediators. Indeed, the ECB system interacts with key hormones in the CNS and the periphery that are known to influence food intake and weight gain. For instance, ECBs are tonic inhibitors of neuropeptides such as CCK and corticotrophin releasing hormone (CRH), most probably via co-localization of CCK and CRH receptors with CB_1_ receptors [131,132]. CCK is a peptide produced by the pancreas and a neurohumoral agonist acting to enhance insulin secretion. It was also proven that ECBs have orexigenic effects, which mean that they are high when fasting and low while eating, and these effects could be related to ECBs’ interactions with leptin and ghrelin. Interestingly, it has been shown in rodents that leptin, a peptide hormone produced by fat cells and involved in weight control and metabolism, decreases ECBs levels in the hypothalamus [130]. Complementarily, a tonic elevation of ECBs has been linked in rodents with an increase in plasmatic ghrelin, a peptide hormone regulating appetite, suggesting that the orexigenic effects of ECBs could be ghrelin-dependent. Finally, it must be mentioned that ECBs influence weight gain via peripheral metabolic mechanisms. There is indeed evidence showing that anandamide regulates adipocyte differentiation [133] and that adipose cells generate ECBs, express CB_1_ receptors and respond to CB_1_-induced lipogenesis [134] and fatty acid synthesis [135]. Although preliminary, the results of human studies are generally consistent with these pre-clinical findings. For instance, pharmacological trials have shown that rimonabant—a CB_1_ antagonist—is efficient for the treatment of obesity [136]. Preliminary genetic association studies also suggest an involvement of the ECB system in obesity and/or metabolic disorders [137]. Overall, current evidence suggests that a hyperactivity of the ECB system could contribute to obesity development and probably to the metabolic syndrome, but further confirmation of this assumption is required in humans.

The metabolic syndrome is widely prevalent in individuals with schizophrenia, who are more prone to develop diabetes, obesity and dyslipemia than the general population. Schizophrenia patients have 20% shorter life expectancy than that of the general population [138]. Metabolic disorders contribute significantly to this epidemiological burden of schizophrenia, and are associated with poor quality of life and antipsychotic non-compliance [139,140]. Atypical antipsychotics have been demonstrated to exacerbate this problem. However, evidence is cumulating to demonstrate that the schizophrenia diagnosis increases the risk for metabolic disorders independently of environmental exposure such as inactivity, smoking and dietary habits, even when pharmacological treatment and lifestyle are considered as covariates [141].

Based on these findings, we hypothesized that the ECB system could be involved in the predisposition of schizophrenia patients to develop metabolic disorders. Indeed, central and peripheral ECB disturbances have been demonstrated in patients with schizophrenia. In addition, some of the endocrinal factors contributing to weight gain in schizophrenia are known to be influenced by the ECB system. Schizophrenia patients seem to have higher fasting cortisol and fasting insulin and lower insulin-like growth factor-1, relative to controls [142]. There is also impaired glucose tolerance in first-episode drug-naive patients with schizophrenia [143]. However, no significant differences are observed on any lipid measure, leptin, HbA1C or fasting glucose between schizophrenia patients and controls [141]. Knowing the interaction between CCK and insulin and the obvious role of insulin in glucose metabolism, the study of the ECB system could reveal an explanation for weight gain in schizophrenia and its underlying physiopathology which remains unknown. 

From a therapeutic perspective, agents acting on the ECB system have been proposed for the treatment of both patients with schizophrenia and patients with metabolic disorders. Interestingly, while CB_1_ receptor antagonists, such as rimonabant, are known for their antipsychotic and anti-obesity effects, they seem also to activate PPAR-γ, suggesting that their effects may not be mediated via CB_1_ receptors [144] but rather by a receptor involved in metabolism. Even more relevant, among anti-diabetic agents currently used, there is thiazolidinedione pioglitazone, which acts on the nuclear transcription factor PPARγ, increasing insulin sensitivity and causing a significant reduction in free fatty acid levels [145]. This leaves open the possibility to combine treatment for patients with schizophrenia suffering from metabolic problems but it allows the study of the schizophrenia-weight gain from a physiopathological perspective. Moreover, PPAR ligands (PEA and OEA) have been explored in patients with schizophrenia. OEA is an anorexic lipid that produces satiety and reduces weight gain in rodents [146]. Plasmatic OEA was increased in patients relative to controls, even though patients in this sample did not significantly differ in weight compared with controls [100]. This paradoxical result may be influenced by substance abuse and further studies are needed. 

Overall, futures studies on the ECB system in schizophrenia might produce new insights about the complex biological relationships between schizophrenia and metabolic disorders, which are highly prevalent in the disorder and a source of significant burden.

### 5.2. Endocannabinoids and drug reward

Mounting evidence suggests that the ECB system plays a critical role in the brain reward system [147]. CB_1_ receptors are present in ventral tegmental area and the nucleus accumbens, the key brain reward regions, as well as in the prefrontal cortex, the amygdala and hippocampus, which are interconnected with the brain reward circuitry [148]. A common neurobiological mechanism underlying drug abuse involves ECB release in the ventral tegmental area, causing a rewarding effect and increasing the motivation to seek drugs. The primary rewarding effects of cannabinoids, opioids, nicotine and alcohol increase dopaminergic firing rates and depend on ECB release in the ventral tegmental area [149]. In contrast, psychostimulants enhance dopamine in nucleus accumbens directly on axon terminals, without necessitating the activation of CB_1_ receptors [58]. Thus, the rewarding effects of alcohol, cannabinoids, nicotine and alcohol may be dependent on the ECB system, but not those of psychostimulants. This role in plasticity is important for learning processes related to addictive behaviour, and it seems to be dopamine-independent. Consistent with these preclinical findings, preliminary results from human studies have shown an association between homozygous form of FAAH gene 385C/A polymorphism and alcohol/drug abuse in a Caucasian population [150]. Similarly, associations have been described between alcohol and drug abuse/dependence and various polymorphisms of the *CNR1* gene [151,152,153,154]. Genomic variants at the CB_1_/Cnr1 locus are candidates for human vulnerability to substance use disorders. A genomic study of human CB_1_/*Cnr1* found four exons that may thus represent CB1/Cnr1 genomic structure expressed in regions that include hippocampus, cerebellum, amygdala, caudate putamen and substantia nigra [155].This study has begun to elucidate candidate promoter regions for the CB1/Cnr1 gene and also variants in genomic sequences, such as pattern of CB1/Cnr1 transcriptional start and splice variants that might confer CB1/Cnr1 regulation. Furthermore, there seems to be haplotypes toward the 5’ end of the CB1/Cnr1 gene’s exons and introns distinguishing substance abusers from control individuals. Addiction-associated TAG haplotype is associated with significantly reduced expression of CB1/Cnr1 exon 3 mRNA in human brain. Lastly, allelic differences in CB1/Cnr1 expression and regulation are good candidates to play roles in producing differences in addiction vulnerabilities. In view of these results, therapeutic alternatives involving ECBs are growingly considered for drug abuse treatment. For example, CB_1_ receptor activation or deactivation have been proposed, but there remains many concerns about potential adverse emotional reactions [156].

The key feature of (nearly) all addictive drugs is their ability to increase synaptic dopamine levels in the striatum. Regarding Δ^9^-THC, its exact effects on brain reward are still controversial. CB_1_ receptor mediates cannabis effects in the CNS [156,157]. While binding CB_1_ receptors, Δ^9^-THC acts as a functional antagonist interacting with ECBs, anandamide and 2-AG, which usually inhibit neurotransmission. More precisely, Δ^9^-THC seems to be a CB_1_ receptor partial agonist, acting as an antagonist in presence of high efficacy ECBs, like 2-AG, and as an agonist in presence of low efficacy ECBs, like anandamide [158]. In seven healthy subjects, it was recently shown, using PET, that Δ^9^-THC induces dopamine release in the ventral striatum and the pre-commissural dorsal putamen, but not in other striatal sub-regions [159]. Interestingly, a recent PET study showed that the dopaminergic hyperactivity underlying the psychotic symptoms of schizophrenia is more prominent in the associative regions of the striatum, such as the pre-commissural dorsal caudate region [160]. However, it must be mentioned that some results challenge the notion that striatal dopaminergic hyperactivity underlies the association between cannabis smoking and psychotic outcomes. Indeed, a PET study involving thirteen healthy recreational cannabis users failed to show that 10 mg of Δ^9^-THC releases significant amounts of striatal dopamine, compared to placebo [161].

Substance use disorders are highly prevalent in schizophrenia and are associated with significant psychiatric and functional impairments [162]. Given the importance of this comorbid disorder, Leweke et al. (2007) examined the influence of cannabis smoking on the CSF anandamide levels of schizophrenia patients [163]. Individuals with schizophrenia and low cannabis consumption had CSF anandamide levels ten times higher than schizophrenia individuals consuming more cannabis or healthy controls (with or without cannabis consumption). There was no difference in plasmatic anandamide levels between the four sub-groups. Moreover, CSF anandamide levels were inversely correlated with positive symptoms, regardless of consumption. These results suggest that among individuals with schizophrenia, frequent exposure to cannabis reduces anandamide signalling in the CNS (via reduced synthesis or accelerated degradation), a phenomenon not observed in healthy subjects. Given the key role of ECBs in the brain reward system, preliminary evidence suggests that ECBs contribute to the propensity of schizophrenia patients to abuse psychoactive substances, especially cannabis and alcohol. Noticeably, our group recently showed that baseline plasma anandamide levels predict substance consumption (alcohol and cannabis) after twelve weeks of atypical antipsychotic treatment, quetiapine, in patients with schizophrenia and comorbid substance abuse [100]. 

Interestingly, electrophysiology studies in rats have shown that ECB elevation via FAAH inhibition suppresses nicotine-induced activation of ventral tegmental area dopamine neurons via PPAR-α [164]. In other words, anandamide inhibits mesolimbic dopamine in drug reward via PPAR. However, intracerebral anandamide analog infusion did not produce this effect, suggesting that PEA or OEA may play a role in drug reward. Consistent with this view, our group showed an elevation of plasma OEA levels in patients with schizophrenia and substance use disorders, relative to healthy controls [100]. Furthermore, ECBs facilitate effects of orexin-releasing neurons in hypothalamus, which also project to the nucleus accumbens and ventral tegmental area. Hypothalamus orexins are involved in the rewarding effects of psychoactive substances [58]. Interestingly, drug craving is quite similar with food craving observed in patients with bulimia or obesity problems. In the future, it will be of interest to examine the potential roles of the ECB system in food or drug craving in schizophrenia.

## 6. Conclusions

The ECB system is involved in several neuromodulation processes. Pre-clinical studies strongly suggest that ECBs play a role in neurogenesis, neurodegenerative processes, as well as in the neural circuits thought to be impaired in schizophrenia. In humans, mounting evidence shows that CB_1_ receptor densities are altered in schizophrenia and that anandamide levels are elevated, suggesting that the ECBs are involved in the pathophysiology of the disorder. Given the key roles of ECBs in drug reward and metabolic regulation, it can be hypothesized that ECBs may be involved in some worrying comorbidities associated with schizophrenia, such as metabolic disorders and substance abuse/dependence. Novel pharmacologic perspectives for the treatment of schizophrenia are increasingly emphasizing the potential applications of the ECB system, which will need to be tested in well-controlled trials. Further studies controlling for substance abuse are needed to better understand the roles of the ECB system in schizophrenia. 
